# Virus-Induced Pathogenic Antibodies: Lessons from Long COVID and Dengue Hemorrhage Fever

**DOI:** 10.3390/ijms26051898

**Published:** 2025-02-22

**Authors:** Der-Shan Sun, Te-Sheng Lien, Hsin-Hou Chang

**Affiliations:** Department of Molecular Biology and Human Genetics, Tzu-Chi University, Hualien 970, Taiwan; dssun@mail.tcu.edu.tw (D.-S.S.); alan211@mail.tcu.edu.tw (T.-S.L.)

**Keywords:** virus-induced pathogenic antibodies, antibody-dependent enhancement, post-acute sequelae of SARS-CoV-2 infection, dengue hemorrhagic fever, immune dysregulation, autoantibodies, chronic inflammation

## Abstract

Virus-induced antibodies represent a dual-edged sword in the immune response to viral infections. While antibodies are critical for neutralizing pathogens, some can paradoxically exacerbate disease severity through mechanisms such as antibody-dependent enhancement (ADE), autoantibody, and prolonged inflammation. Long coronavirus disease (COVID) and dengue hemorrhagic fever (DHF) exemplify conditions where pathogenic antibodies play a pivotal role in disease progression. Long COVID is associated with persistent immune dysregulation and autoantibody production, leading to chronic symptoms and tissue damage. In DHF, pre-existing antibodies against dengue virus contribute to ADE, amplifying viral replication, immune activation, and vascular permeability. This review explores the mechanisms underlying these pathogenic antibody responses, highlighting the shared pathways of immune dysregulation and comparing the distinct features of both conditions. By examining these studies, we identify key lessons for therapeutic strategies, vaccine design, and future research aimed at mitigating the severe outcomes of viral infections.

## 1. Introduction

Viruses pose a significant challenge to the human immune system, triggering complex host responses aimed at neutralizing and clearing the pathogen. Among these responses, pathogenic antibody production is a critical element of the adaptive immune system, serving as a primary defense mechanism [1,2]. While most antibodies are protective and play a vital role in viral clearance [3,4,5], certain viral infections can elicit antibodies that paradoxically contribute to disease progression [6]. These so-called “pathogenic antibodies” can exacerbate infection severity through mechanisms such as antibody-dependent enhancement (ADE), immune complex formation, autoimmunity, and sustained inflammation [6,7]. Understanding these phenomena is essential for advancing our knowledge of viral immunopathology and improving strategies for the prevention and management of severe viral diseases.

In recent years, long coronavirus disease (COVID) and severe dengue (also known as dengue hemorrhagic fever, DHF) have emerged as significant examples of conditions where virus-induced pathogenic antibodies may play a critical role [8,9]. Long COVID (also known as post-acute sequelae of SARS-CoV-2 infection, post-COVID syndrome, and post-acute sequelae of COVID-19), a condition characterized by persistent symptoms following infection with severe acute respiratory syndrome coronavirus 2 (SARS-CoV-2), the virus responsible for coronavirus disease 2019 (COVID-19), manifests through symptoms such as fatigue, cognitive dysfunction, and cardiovascular abnormalities [10,11,12,13,14,15,16,17,18,19,20,21,22,23,24,25,26,27,28,29,30,31,32]. These symptoms are believed to result from immune dysregulation, including chronic inflammation, autoantibody production, and sustained immune activation, accompanied by dysregulated antibody responses [8,9]. Similarly, DHF, a severe manifestation of dengue virus (DENV) infection, is strongly linked to ADE and autoantibodies, where pre-existing antibodies worsen disease severity upon subsequent infections [7,33,34,35]. Broadly speaking, similar to long COVID, DHF, a severe form of dengue which may lead to mortality during secondary DENV infections, could also be considered as a syndrome arising after a primary DENV infection (post-DENV primary infection syndrome).

This review aims to explore the mechanisms by which virus-induced antibodies can become pathogenic, with a focus on insights gained from studying long COVID and DHF. By examining these two conditions, we seek to highlight the common pathways of immune dysregulation, discuss the implications for therapeutic and vaccine development, and identify areas for future research. Ultimately, a deeper understanding of these processes will provide critical lessons for managing viral infections and mitigating their long-term impacts on human health.

## 2. Background on Virus-Induced Antibodies and Their Role in Pathogenesis

Antibodies are specialized proteins produced by B cells in response to the presence of antigens, such as viral proteins [1,2]. They play a critical role in the immune system by neutralizing pathogens, marking infected cells for destruction, and preventing viral replication. This protective role of antibodies is central to the success of vaccines and natural immunity [1,2]. However, in certain viral infections, antibodies intended to protect the host can instead drive pathogenic processes through mechanisms like ADE and the induction of autoimmunity [7,8,9,33,34,35].

**Figure 1:**
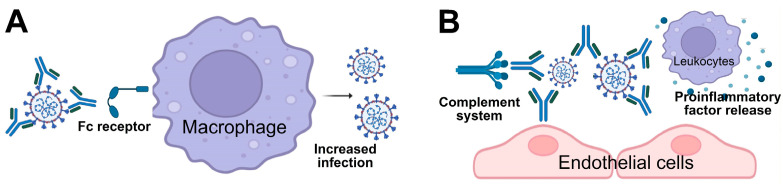
Two proposed mechanisms of ADE in viral disease exacerbation. This figure illustrates two potential mechanisms of ADE contributing to viral disease pathogenesis, as suggested by previous studies [37,40]. (**A**) In ADE via increased infection, non-neutralizing or sub-neutralizing antibodies enhance the viral infection of macrophages or other Fc-receptor-bearing cells through Fc-receptor-mediated endocytosis. This process leads to increased viral replication and a more severe disease phenotype. (**B**) In ADE via enhanced immune activation, non-neutralizing antibodies form immune complexes with viral antigens within tissues such as blood vessels or airways. These immune complexes trigger the release of proinflammatory cytokines, the recruitment of immune cells, and the activation of the complement cascade, resulting in localized tissue damage and inflammation. Figure created with BioRender.com.

Classical ADE is a well-known mechanism of antibody-mediated pathogenicity, where non-neutralizing or suboptimal antibodies bind to a virus, forming immune complexes that are internalized by Fc-receptor-bearing cells such as monocytes and macrophages [7,33,34,35]. This enhances viral replication, immune hyperactivation, and increased tissue damage (Figure 1A). Observed in infections like DENV, ADE is a potential contributor to severe disease manifestations and poses challenges for vaccine development. For instance, vaccine-induced ADE has been linked to setbacks in large-scale dengue vaccine use [34], highlighting its importance as a pathogenic factor in both disease progression and vaccine design.

Another mechanism involves the formation of immune complexes—aggregates of antibodies bound to viral antigens. Unlike classical ADE, which enhances viral replication, these immune-stimulating virus–antibody complexes can accumulate in tissues, initiating inflammatory cascades that lead to tissue damage and organ dysfunction [36,37]. This process has been implicated in various viral diseases, including COVID-19 [36,37,38,39]. Recent studies have expanded the definition of ADE in the context of SARS-CoV-2 pathogenesis [36,37], describing it more broadly as an antibody-dependent enhancement of pathogenesis. In this framework, immune-complex-induced immune activation is recognized as an alternative ADE pathway that amplifies immune responses in COVID-19 (Figure 1B). The current evidence highlights immune complex formation, complement deposition, and localized immune activation as contributors to ADE in COVID-19. This mechanism—where virus–antibody complexes worsen disease progression—is increasingly regarded as a distinct form of ADE [36,37] (Figure 1B). These processes, occurring within airways or vascular tissues, contribute to inflammation and tissue damage, emphasizing the complexity of ADE in the context of SARS-CoV-2 (Figure 1B).

Various mechanisms have been implicated in the virus-induced production of pathogenic antibodies, including epitope spreading, molecular mimicry, bystander activation, and original antigenic sin [41,42,43]. These processes can give rise to antibodies that are non-neutralizing against the virus or cross-react with host tissues, leading to abnormal immune activation, chronic inflammation, and tissue damage. In COVID-19, the precise mechanisms driving autoantibody production remain unclear. However, several studies have identified a diverse range of autoantibodies in patients with acute COVID-19 [44,45]. For example, high-affinity SARS-CoV-2-neutralizing antibodies have been found to cross-react with mammalian self-antigens in the gut, kidney, lung, heart, and brain. In the brain, antibody binding has been observed in regions such as the basal ganglia, hippocampal formation, olfactory bulb, and cerebral cortex [45]. Another study reported that SARS-CoV-2 proteins share homology with neuronal protein epitopes located within the vagus nerve and brainstem nuclei, including the jugular ganglion, nodose ganglion, dorsal motor nucleus, and nucleus ambiguus [46]. In the context of long COVID, antibody responses can persist long after the acute infection has been resolved. These sustained autoantibody responses have been linked to prolonged inflammation, vascular damage, and a wide range of chronic symptoms and tissue injury [39]. Accordingly, several potential mechanisms have been proposed, including the following: (1) Molecular mimicry: Structural similarities between SARS-CoV-2 proteins and human proteins may cause the immune system to mistakenly target host tissues, leading to autoimmunity [46]; (2) Persistent viral antigens: Viral fragments that remain in the body post-infection can continuously stimulate the immune system, promoting the production of pathogenic antibodies over time [27]; (3) Immune dysregulation: SARS-CoV-2 infection can disrupt immune homeostasis, leading to the bystander activation of leukocytes and the production of abnormal antibodies that attack healthy tissues [27,47]. Although the exact mechanisms are still being investigated, the presence of dysregulated antibody responses in long COVID underscores the potential for antibodies to shift from protective to pathogenic roles.

Although both DENV and SARS-CoV-2 are linked to the distinct forms of antibody-enhanced pathogenesis that contribute to disease severity, their underlying mechanisms are different. In DENV infections, virus-induced pathogenic antibodies worsen the disease by facilitating viral entry and replication, whereas in SARS-CoV-2 infections, pathogenic antibodies primarily drive disease progression through excessive immune activation (Figure 1; see references [27,37,40,48]). Understanding these distinct mechanisms is crucial for developing effective therapies and vaccines. By unraveling the factors driving pathogenic antibody responses, researchers can devise strategies to mitigate their detrimental effects while preserving their protective roles. This section provides the groundwork for examining specific case studies of long COVID and DHF, where virus-induced antibodies have been shown to play critical roles in disease progression.

## 3. COVID and Long COVID

COVID-19, caused by severe acute respiratory syndrome coronavirus 2 (SARS-CoV-2), emerged in late 2019 and rapidly escalated into a global pandemic [49,50]. The virus primarily spreads through respiratory droplets and binds to ACE2 receptors on human cells, facilitating viral entry and replication [51]. This interaction enables the virus to induce a wide range of symptoms, from mild lung inflammation and damage to respiratory distress, severe pneumonia, and multi-organ failure [49,51,52,53,54,55,56,57,58,59,60]. Therapeutic approaches have evolved to include antiviral medications, monoclonal antibodies, and supportive care. Vaccination has since become a cornerstone in controlling the pandemic, significantly reducing severe illness and transmission rates [54,61,62,63,64,65,66].

While the acute phase of COVID-19 brought immense challenges, the emergence of long COVID has added a new layer of complexity to the pandemic. Long COVID, also referred to as “post-acute sequelae of COVID-19”, is characterized by persistent symptoms lasting weeks or months after the acute phase of infection has been resolved [27,31,52,67]. Affecting individuals across all ages and severities of initial infection, it presents with multisystemic manifestations, including fatigue, cognitive dysfunction, dyspnea, and cardiovascular abnormalities [10,11,12,13,14,15,16,20,31].

An estimated 10–30% of non-hospitalized cases, 50–70% of hospitalized cases, and 10–12% of vaccinated cases develop long COVID, affecting at least 65 million individuals globally. However, the true number is likely higher due to undocumented cases [27]. Most cases occur in individuals with mild acute illness, particularly among those aged 36–50 years, who represent the majority of documented COVID-19 cases. This condition has been associated with significant new-onset disorders, including cardiovascular, thrombotic, and cerebrovascular diseases, type 2 diabetes, myalgic encephalomyelitis/chronic fatigue syndrome (ME/CFS), and dysautonomia, especially postural orthostatic tachycardia syndrome [27,68,69,70,71,72]. Symptoms can persist for years, with ME/CFS and dysautonomia cases often becoming lifelong. This has contributed to labor shortages, as many affected individuals are unable to return to work [27].

Emerging evidence indicates that immune dysregulation is a central driver of long COVID, involving chronic inflammation and the production of pathogenic antibodies [10,11,12,13,14,15,16,20,31]. Studies have found elevated levels of autoantibodies targeting key receptors, such as ACE2 (the primary receptor for SARS-CoV-2), β2-adrenoceptors, muscarinic M2 receptors, angiotensin II AT1 receptors, and angiotensin 1–7 MAS receptors [73,74,75]. Other autoantibodies target connective tissue, extracellular matrix components, the vascular endothelium, coagulation factors, platelets, and various organ systems, including the lungs, central nervous system, skin, and gastrointestinal tract. Autoantibodies also impact immunomodulatory proteins like cytokines, chemokines, and complement components [27,44].

A primary mechanism implicated in long COVID is the persistence of autoantibodies [31,32,76]. Studies have identified various autoantibodies targeting self-antigens, including components of the vascular endothelium, connective tissues, and immune regulators [31,32]. Although the precise mechanisms remain unclear, these autoantibodies may arise through processes such as molecular mimicry, persistent viral antigen stimulation, and immune dysregulation [27,46,47], as discussed in the previous section. In these cases, antibodies generated against viral antigens cross-react with host tissues, triggering abnormal immune activation, chronic inflammation, and tissue damage [41,42,43]. For example, autoantibodies targeting ACE2, the receptor for SARS-CoV-2, have been linked to vascular and endothelial dysfunction observed in both acute SARS-CoV-2 infection [75,77,78,79,80,81] and long COVID patients [31,82]. Moreover, the persistence of certain autoantibodies, such as anti-U1-snRNP, anti-SS-B/La, and CHRM3 autoantibodies, in plasma has been linked to specific post-COVID symptoms, including fatigue and dyspnea [83,84]. Meanwhile, ADE has raised concerns in the context of COVID-19 [37,40,85]. These ADE-associated antibodies may enhance viral-induced over-activation of immune cells, contributing to the hyperinflammation and tissue damage of COVID-19, which can worsen disease outcomes [37,40,85]. Although the role of ADE in long COVID is less well-characterized, limited evidence suggests that ADE-associated antibodies, such as anti-nucleocapsid antibodies, may contribute to disease pathogenesis primarily through immune activation (mechanism illustrated in Figure 1B) [85,86,87].

Another factor contributing to long COVID is prolonged immune system activation [76,88]. Persistent viral antigens or RNA fragments may drive chronic immune responses, sustaining the production of pathogenic antibodies and potentially leading to long COVID [22,23,27,89,90]. These antibodies, induced by lingering viral components, may form immune complexes in both acute SARS-CoV-2 infection [38,39,91] and long COVID patients [16]. The deposition of these immune complexes in tissues can activate inflammatory pathways, worsening symptoms and contributing to organ damage (Figure 1B).

The implications of these findings are critical for both diagnosis and treatment. Identifying biomarkers of pathogenic antibody responses, such as specific autoantibodies or immune complexes, could aid in diagnosing long COVID and stratifying patients based on disease severity. Therapeutic strategies aimed at targeting pathogenic antibody responses, such as intravenous immunoglobulin treatment, which is widely recognized for its anti-inflammatory and immunomodulatory effects, show promise in alleviating symptoms and enhancing patient outcomes [29,92,93,94,95,96,97].

## 4. Dengue and Severe Dengue

DENV is a mosquito-borne flavivirus with four distinct serotypes (DENV-1, DENV-2, DENV-3, and DENV-4), capable of causing a wide range of clinical manifestations, from mild dengue fever (DF) to severe dengue (also known as dengue hemorrhagic fever, DHF), as defined by the 2009 World Health Organization classification [41,98,99,100,101,102,103]. While primary DENV infections are typically associated with DF, severe dengue is more frequently observed in secondary infections with a different DENV serotype [41,98,99,100,101]. This phenomenon is attributed to pathogenic immune responses, potentially ADE and antibody production. The complex interplay between DENV serotypes and ADE, and DENV-elicited autoantibodies, highlights the importance of developing targeted vaccine strategies and therapeutic interventions to reduce the risk of DHF [104,105,106,107,108,109,110,111,112,113,114].

Similar to COVID-19, the role of pathogenic antibodies in DHF extends beyond ADE. Studies have also identified anti-DENV autoantibodies that cross-react with host tissues, contributing to the pathogenesis of the disease [115,116]. Various DENV proteins have been shown to induce autoantibodies in animal models. Among these, the DENV nonstructural protein 1 (NS1) exhibits unique biological properties and is capable of eliciting diverse fractions of autoantibodies [117,118,119,120,121,122,123]. These autoantibodies target critical components of the vascular endothelium and coagulation pathways, exacerbating vascular permeability and heightening the risk of hemorrhage. For instance, plasma leakage, a hallmark manifestation of DHF, has been linked to autoantibodies against endothelial cells, which disrupt the integrity of the endothelial barrier [120,124,125,126,127,128,129].

While classical ADE has been widely recognized as a key mechanism in dengue immunopathogenesis, some aspects of its role in severe dengue remain to be fully elucidated. First, despite decades of research, classical ADE (Figure 1A) has been observed in over a dozen viruses, including Ebola, human immunodeficiency virus, Japanese encephalitis, Murray Valley encephalitis, yellow fever, Ross River, Lassa, Rift Valley fever, rabies, polio, coxsackie, hepatitis virus, and herpes simplex [130,131,132,133,134,135,136,137,138]. Given that ADE has been observed across multiple viral infections, a thought-provoking question arises: if ADE-mediated antibody responses during secondary infections were solely responsible for driving severe disease, why do most of these viruses not consistently lead to more severe illness upon reinfection? DENV appears to be unique in its strong association between secondary infection and severe disease, suggesting that while ADE plays a significant role, additional factors may also contribute to the pathogenesis of DHF.

Secondly, if the primary pathogenic effect of classical ADE is to enhance viral replication and increase viral load, and if DHF were solely driven by ADE, one might expect disease severity to peak when viremia is at its highest. However, clinical observations do not fully align with this expectation. Instead, acute DHF typically manifests after fever subsides and viremia declines, coinciding with rising levels of virus-induced antibodies [139]. This discrepancy raises the possibility that mechanisms beyond classical ADE may also be involved in DHF pathogenesis.

One emerging hypothesis that may help explain this complex disease progression is the “two-hit” model, which proposes that DENV infection and autoantibody responses act through distinct but complementary mechanisms to drive severe disease. In this model, the first “hit” is the viral infection itself, where high viremia triggers inflammatory responses and cellular dysfunction. This effect is partly attributed to the DENV virion or its envelope protein domain III (EIII), a structural component involved in host cell entry. Recent studies suggest that the DENV virion or EIII alone can activate the NLRP3 inflammasome, leading to platelet, leukocyte, and endothelial activation in mice. However, this activation alone does not appear to be sufficient to cause hemorrhagic manifestations [119,122,140,141].

The second “hit” involves the induction of autoantibodies, which may be triggered by viral proteins such as NS1 [117,118,119,120,121,122,123], and arise alongside antiviral antibodies during the immune response to DENV infection. These autoantibodies differ from neutralizing anti-virion or anti-EIII antibodies and instead target host proteins, such as platelet, leukocyte, and endothelial cell antigens [117,118,119,120,121,122,123]. Unlike protective antiviral antibodies that aid in viral clearance, cross-reactive autoantibodies may contribute to immune dysregulation, tissue damage, and systemic inflammation [117,119,120,121,122,123].

A valid concern regarding the two-hit model is why a secondary DENV infection appears necessary for a more severe disease phenotype if both the “first hit” and “second hit” contribute to tissue damage. Insights from in vivo studies may help address this question. Experimental models have shown that mice treated with DENV (or EIII at DHF-equivalent viral loads, representing the first hit) or autoantibodies (the second hit) alone exhibit inflammatory activation but do not develop hemorrhagic manifestations or lethality. However, when anti-platelet autoantibodies are administered 24 h after EIII treatment, the inflammatory response is significantly amplified, leading to hemorrhage and mortality [119,122]. Clinically, severe dengue often follows a biphasic course: early infection is characterized by peak viremia, followed by a critical phase where the viral load declines, antibody titers rise, and vascular leakage and hemorrhagic symptoms emerge [101]. This pattern aligns with the two-hit model, in which virus-induced inflammation primes the immune system, and subsequent autoantibody-mediated effects drive severe disease progression [119,120,122,123].

**Figure 2:**
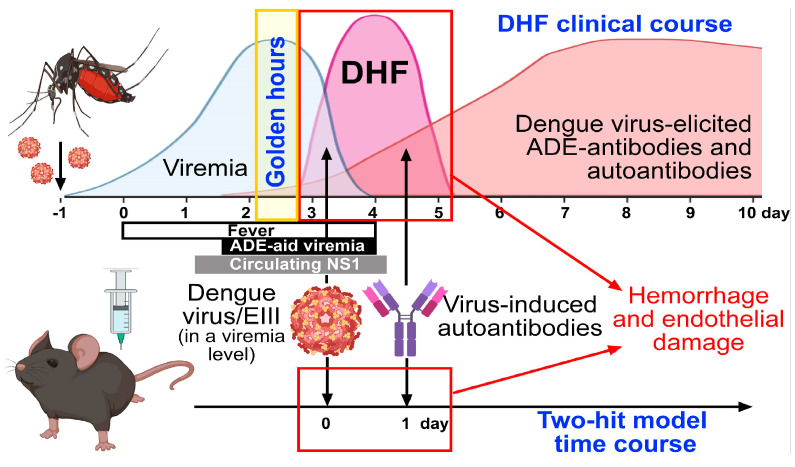
The schematic illustration aligns the clinical progression of severe dengue (also known as dengue hemorrhagic fever, DHF) with the two-hit mouse model. Based on the reported timeline of severe dengue manifestations [101,142], a conceptual two-hit model was proposed. In this timeline, day “−1” represents the mosquito bite, while day 0 marks the onset of fever. Figure created with BioRender.com.

Accordingly, rather than downplaying the crucial role of ADE in dengue pathogenesis, the two-hit hypothesis offers a complementary perspective that, alongside ADE, may help clarify some of the unresolved questions about disease progression. A proposed model integrating both ADE and the two-hit hypothesis is illustrated in Figure 2. Further investigations into these mechanisms are warranted, as they will offer deeper insights into the interplay between DENV infection, ADE, and pathogenic antibody responses in severe dengue.

In the two-hit model, the first “hit” in severe dengue involves the cell-damaging viral factors, such as circulating DENV, virion-associated EIII, and circulating NS1. The viral factors, such as EIII and NS1, are known to interact with and exert toxic effects on various cell types [118,122,123,140,141,143,144,145,146,147,148]**,** making them potential contributors for the first hit. In parallel, ADE can elevate viremia by enhancing viral infectivity in Fc-receptor-expressing leukocytes, leading to an increased presence of the circulating virus and virion-associated EIII, thereby intensifying the impact of the first hit (Figure 2, upper panel, black box: ADE-aid viremia).

The second “hit” occurs when the host immune response generates autoantibodies alongside antiviral antibodies. These autoantibodies can target host cells, contributing to immune-mediated tissue damage and vascular dysfunction (Figure 2, upper panel). Our previous studies demonstrated that, in a reductionist approach, EIII alone is sufficient to activate inflammatory responses but does not cause hemorrhagic pathogenesis. However, in two-hit mouse models, exposure to EIII followed by the administration of autoantibodies leads to target cell damage, including endothelial apoptosis and hemorrhage (Figure 2, lower panel) [119,120,122,147,148].

This model suggests the existence of a critical time window—referred to as the “golden hours” (highlighted in yellow) during which viremia (EIII levels) peaks while autoantibody production has not yet reached levels that cause cellular damage (Figure 2, upper panel). Since severe complications and high mortality in dengue patients often arise as fever subsides and viremia declines [139], there is a risk that high-risk patients may be discharged too early from the hospital. Identifying patients with both high autoantibody levels and persistent DENV-induced cellular damage within this hypothetical time window may help prioritize intensive monitoring and early intervention, potentially improving patient outcomes.

These findings have implications for both vaccine development and therapeutic interventions. Current dengue vaccines must carefully balance achieving protective immunity while minimizing the risk of ADE, autoantibody generation, and associated safety concerns [34,149,150,151]. Therapeutic strategies targeting the downstream effects of pathogenic antibodies, such as anti-inflammatory treatments, may prove useful in reducing disease severity [152,153,154,155]. Furthermore, animal studies suggest that identifying biomarkers of autoantibody production could aid in early diagnosis and risk stratification for patients at risk of developing DHF [119,122]. An optimal time point for such early diagnosis, as illustrated in Figure 2 (the hypothetical “golden hours”), might be instrumental in screening patients with a higher risk of progressing to DHF.

## 5. Comparison of Pathogenic Mechanisms in Long COVID and Dengue

While long COVID and DHF are caused by distinct viruses—SARS-CoV-2 and DENV, respectively—both conditions illustrate how virus-induced pathogenic antibodies can play pivotal roles in disease progression. A comparative analysis of their underlying mechanisms reveals several similarities and differences that enhance our understanding of immune dysregulation in viral infections (Table 1).

## 6. Conclusions

Virus-induced pathogenic antibodies highlight the delicate balance between protective and harmful immune responses in viral infections. Long COVID and DHF provide compelling examples of how dysregulated antibody responses can drive chronic inflammation, tissue damage, and severe disease outcomes. While long COVID highlights the role of persistent autoantibodies and prolonged immune activation, DHF exemplifies the impact of ADE and autoantibody-induced vascular dysfunction. Understanding these mechanisms offers critical insights into designing safer vaccines and targeted therapies that mitigate the risk of pathogenic antibody responses while preserving protective immunity. Future research should focus on identifying the biomarkers of pathogenic antibody activity, exploring immunomodulatory treatments, and developing vaccines that minimize ADE and autoantibody generation. These efforts will be essential for improving clinical outcomes and reducing the long-term impacts of viral infections on global health.

## Figures and Tables

**Table 1:** Comparison of the potential roles of virus-induced pathogenic antibodies in long COVID and DHF.

Aspect	Long COVID	Dengue Hemorrhagic Fever (DHF)	Similarities	Differences
Pathogenic Mechanism	Persistent autoantibodies targeting host tissues [31,32,75,77,78,79,80,81,82].	Autoantibodies targeting the vascular endothelium and coagulation pathways [115,116,120,124,125,126,127,128,129].	Pathogenic antibodies play a central role in driving immune dysregulation and tissue damage [6,7,31,32,75,77,78,79,80,81,82,115,116].	Long COVID involves systemic immune dysregulation, while DHF is strongly linked to vascular permeability and hemorrhagic features [31,32,75,77,78,79,80,81,82,104,105,106,107,108,109,110,111,112,113,114].
Antibody-Dependent Enhancement (ADE)	Immune-complex-induced inflammation is likely involved in the pathogenesis of COVID and long COVID [37,40,85].	Well-documented mechanism facilitating viral entry and replication in immune cells [7,156,157,158,159,160].	ADE, viral factors, and autoantibodies may exacerbate disease severity in both conditions [7,37,85,156,157,158,159,160].	ADE, viral factors, and autoantibodies are likely primary drivers in DHF, whereas immune complexes are likely involved in the pathogenesis of COVID and long COVID [7,37,85,156,157,158,159,160].
Autoantibody Production	Autoantibodies against self-antigens may contribute to chronic inflammation and damage [20,31,32,75,77,78,79,80,81,82].	Autoantibodies targeting endothelial cells and other structures disrupt vascular integrity [115,116,120,124,125,126,127,128,129].	Autoantibodies contribute to immune dysregulation and tissue damage in both conditions [115,116,120,124,125,126,127,128,129].	Autoantibodies in long COVID are associated with systemic symptoms, while in DHF, they are more localized to the vasculature [31,32,75,77,78,79,80,81,82,115,116,120,124,125,126,127,128,129].
Inflammation	Prolonged immune activation driven by viral RNA/antigen persistence [16,17,18,19,21,22,23,38,39,91].	Acute inflammation potentially amplified by immune complexes and autoantibodies [115,116,120,124,125,126,127,128,129,161,162].	Both exhibit immune dysregulation and inflammation as key contributors to disease progression [6,7,115,116].	Long COVID involves chronic, prolonged inflammation, whereas DHF is characterized by an acute cytokine storm [16,17,18,19,38,39,91,104,105,106,107,108,109,110,111,112,113,114,162].
Clinical Progression	Symptoms chronically persist long after viral clearance, including fatigue, vascular, and multi-organ issues [16,27,31,52,67,82].	Acute hemorrhagic phase typically follows viral clearance, accompanied by plasma leakage [35,101,105,106,108,111].	Disease progression correlates with immune dysregulation following viral clearance [16,27,31,104,105,106,107,108,109,110,111,112,113,114].	Long COVID is chronic, with systemic and multi-organ effects, while DHF manifests acutely with vascular and hemorrhagic symptoms [16,27,31,104,105,106,107,108,109,110,111,112,113,114].
Potential Implications for Therapy	Focus on immunomodulation (e.g., IVIg, plasmapheresis) and mitigation of autoantibody effects [29,92,93,94,95,96,97,163].	Emphasis on preventing ADE, autoantibody pathogenesis, and blocking Fc receptor interactions [119,122,123,125,164].	Strategies aim to target immune dysregulation and reduce the impact of pathogenic antibodies [29,92,93,94,95,96,97,163,164].	Therapies for long COVID target chronic immune dysregulation, while DHF therapies focus on acute vascular leakage and ADE [24,25,26,27,28,29,92,93,94,95,96,97,164].
Vaccine Challenges	Risk of eliciting antibodies that may contribute to pathogenic responses [37,40,85,165].	Balancing immunity with the risk of ADE and autoantibody induction in subsequent infections [7,156,157,158,159,160].	Vaccine development must carefully balance protective immunity with minimizing pathogenic antibody risks [7,85,117,118,119,120,121,122,123,156,157,158,159,160,165].	Long COVID vaccine challenges involve avoiding ADE and promoting durable immunity; dengue vaccine challenges include ADE and serotype effects [7,24,25,26,27,28,85,117,118,119,120,121,122,123,156,157,158,159,160].
